# The improved grasshopper optimization algorithm and its applications

**DOI:** 10.1038/s41598-021-03049-6

**Published:** 2021-12-09

**Authors:** Peng Qin, Hongping Hu, Zhengmin Yang

**Affiliations:** 1 School of Electrical and Control Engineering, North University of China, Taiyuan, 030051 Shanxi China; 2 School of Science, North University of China, Taiyuan, 030051 Shanxi China; 3 Shanxi Key Laboratory of Information Detection and Processing, Taiyuan, 030051 Shanxi China

**Keywords:** Applied mathematics, Computational science

## Abstract

Grasshopper optimization algorithm (GOA) proposed in 2017 mimics the behavior of grasshopper swarms in nature for solving optimization problems. In the basic GOA, the influence of the gravity force on the updated position of every grasshopper is not considered, which possibly causes GOA to have the slower convergence speed. Based on this, the improved GOA (IGOA) is obtained by the two updated ways of the position of every grasshopper in this paper. One is that the gravity force is introduced into the updated position of every grasshopper in the basic GOA. And the other is that the velocity is introduced into the updated position of every grasshopper and the new position are obtained from the sum of the current position and the velocity. Then every grasshopper adopts its suitable way of the updated position on the basis of the probability. Finally, IGOA is firstly performed on the 23 classical benchmark functions and then is combined with BP neural network to establish the predicted model IGOA-BPNN by optimizing the parameters of BP neural network for predicting the closing prices of the Shanghai Stock Exchange Index and the air quality index (AQI) of Taiyuan, Shanxi Province. The experimental results show that IGOA is superior to the compared algorithms in term of the average values and the predicted model IGOA-BPNN has the minimal predicted errors. Therefore, the proposed IGOA is an effective and efficient algorithm for optimization.

## Introduction

Based on the existence of the constraint conditions, optimization models are divided into unconstrained optimization models and constrained optimization models, which widely exist in computer science^1^, artificial intelligence^2^, pattern recognition^3^, energy consumption^4^, truss structural systems^5^, engineering areas^6^, nonlinear time series^7^, and so on.

Swarm intelligence algorithms recently proposed have been applied to solve the optimization models obtained from different actual problems. The genetic algorithm (GA) by Holland in 1992^8^ and the particle swarm optimization (PSO) by Eberhart and Kennedy in 1995^9^ provided some insights for researchers to propose many other swarm intelligence algorithms, such as Moth-fame optimization algorithm (MFO)^6^, Multi-verse optimizer (MVO)^10^, Sine cosine algorithm (SCA)^11^, Whale optimization algorithm (WOA)^12^, Grey wolf optimizer (GWO)^13^, Bird mating optimizer (BMO)^14^, Harris Hawks Optimizer (HHO)^15^, Grasshopper Optimization Algorithm (GOA)^16^, Dragonfly Algorithm (DA)^17^, Slap Search Algorithm (SSA)^18^, Arithmetic Optimization Algorithm (AOA)^19^ and Aquila Optimizer (AO)^20^. These swarm intelligence algorithms have the exploration phase and the exploitation phase. According to the different simulations, every swarm intelligence algorithm has the prominent updated method of the individual and the different applied background. Generally, benchmark functions are applied to test the performance of these swarm intelligence algorithms. Experimental results show that not all swarm intelligence algorithms can solve the whole optimization problems.

Machine learning has been used to solve the prediction and classification problems. For example, Support Vector Regression (SVR) and Long Short-Term Memory (LSTM) based deep learning model were combined to establish the deep learning method for predicting the AQI values accurately, which helped to plan the metropolitan city for sustainable development^21^. And LSTM Recurrent Neural Network was also utilized to preform the stock market prediction^22^. Especially, the randomness of the parameters of machine learning leads to unstable results of predictions and classifications. The fitter are the parameters, the better are the results. Swarm intelligence algorithms can be used to optimize the fitter parameters of machine learning. The problems of optimizing the parameters of neural network by using swarm intelligence algorithm are actually the optimization problem. For example, SCA and GA were used to optimize the parameters of BP neural network for predicting the direction of the stock market indices^23,24^; the improved Exponential Decreasing Inertia Weight-Particle Swarm Optimization Algorithm was utilized to optimize the parameters of generalized radial basis function neural network with AdaBoost algorithm for stock market prediction^25^; least squares support vector machine (LSSVM) with the parameters optimized by the Bat algorithm (BA) was used to forecast the air quality index (AQI)^26^; MVO and PSO algorithms were combined to optimize the parameters of Elman neural network for classification of endometrial carcinoma with gene expression^27^. Swarm intelligence algorithms were used to be hybridized with artificial neural network for predicting the carbonation depth for recycled aggregate concrete^28^. Swarm intelligence algorithms based technique were employed to perform the sparse signal reconstruction^29^.

In particular, GOA proposed in 2017 mimics the behaviour of grasshopper swarms in nature for solving optimization problems^16^. In^16^, GOA was firstly preformed on a set of test problems including CEC2005 qualitatively and quantitatively and then is applied to find the optimal shape for a 52-bar truss, 3-bar truss, and cantilever beam. Then, GOA is improved and is applied into the different fields. A novel periodic learning ontology matching model based on interactive GOA proposed in^30^ considered the periodic feedback from users during the optimization process, using a roulette wheel method to select the most problematic candidate mappings to present to users, and take a reward and punishment mechanism into account for candidate mappings to propagate the feedback of user, which is conducted on two interactive tracks from Ontology Alignment Evaluation Initiative. In^31^, a dynamic population quantum binary grasshopper optimization algorithm based on mutual information and rough set theory for feature selection is performed in twenty UCI datasets. In^32^, GOA was employed to design a linear phase finite impulse response (FIR) low pass, high pass, band pass, and band stop filters. In^33^, GOA was firstly to find optimal parameters with the aim of fusing low-frequency components and then the Kirsch compass operator is used to create an efficient rule for the fusion of high-frequency components, which allows the fused image to significantly preserve details transferred from input images. In^34^, the main controlling parameter of GOA was taken to be a new adaptive function to enhance the exploration and exploitation capability, thus the improved GOA is obtained and then is utilized to optimize the hyperparameters of the support vector regression with embedding the feature selection simultaneously by running on four datasets. In^35^, on the one side, the theoretical perspectives of GOA were given by its versions of modifications, hybridizations, binary, chaotic, and multi-objective; and on the other side, GOA had its applied regions, such as test functions, machine learning, engineering, image processing, network, parameter controller.

The influence of the gravity force and the velocity on GOA and its improvements have not be considered in the basic GOA, which possibly causes GOA to have the slower convergence speed. Based on this, the two updated ways of the position of every grasshopper are proposed in this paper. One is that the gravity force is introduced into the updated position of every grasshopper in the basic GOA. And the other is that the velocity is introduced into the updated position of every grasshopper and the new position are obtained from the sum of the current position and the velocity, which is inspired by PSO. Then every grasshopper adopts its suitable way of the updated position on the basis of the probability. Thus the improved GOA (IGOA) is obtained. Performed on the 23 classical benchmark functions, IGOA is superior to the compared algorithms GOA, PSO, MFO, SCA, SSA, MVO and DA in term of the average values and the convergence speeds. Then, IGOA is tested to optimize the parameters of BP neural network for predicting the closing prices of the Shanghai Stock Exchange Index and the air quality index (AQI) of Taiyuan, thus the predicted model IGOA-BPNN is built. The experimental results show that the IGOA-BPNN has potentiality to optimize the parameters of BP neural network for prediction. Therefore, the proposed IGOA is an effective and efficient algorithm for optimization.

The structure of the paper is organized as follows. The original GOA and the improved GOA are introduced in “Improved grasshopper optimization algorithm”. Section “The function optimization” shows the comparison results of IALO, GOA, PSO, MFO, SCA, SSA, MVO and DA performed on 23 benchmark functions. In “Applications”, IGOA is also utilized to optimize the parameters of BP neural network (BPNN) for predicting the closing prices of the Shanghai Stock Exchange Index and the air quality index (AQI) of Taiyuan, Shanxi Province. Conclusion and discussion are presented in “Conclusions and discussion”.

## Improved grasshopper optimization algorithm

### The basic grasshopper optimization algorithm

GOA proposed in 2017 mimicked the behaviour of grasshopper swarms in nature for solving optimization problems^16^. The mathematical model of simulating the behaviour of grasshopper swarms is as follows^36^:

1 $$X_{i} = S_{i} + G_{i} + A_{i} ,$$

where $X_{i} ,$ $S_{i} ,$ $G_{i} ,$ and $A_{i}$ denote the position, the social interaction, the gravity force and the wind advection of the *i*th grasshopper, respectively. The randomness of the position of grasshoppers is considered and then the Eq. (1) is written to be $X_{i} = r_{1} S_{i} + r_{2} G_{i} + r_{3} A_{i}$, where $r_{1} ,\;r_{2} ,\;r_{3}$ are the random numbers in the interval [0, 1], and $S_{i}$ is defined by

2 $$S_{i} = \sum\limits_{j = 1,j \ne i}^{N} {s(d_{ij} )\hat{d}_{ij} } ,$$

where $N$ denotes the number of the grasshoppers in the swarm, $d_{ij} = \left| {X_{j} - X_{i} } \right|$ is the distance between the *i*th grasshopper and the *j*th grasshopper, $\hat{d}_{ij} = \frac{{X_{j} - X_{i} }}{{d_{ij} }}$ is the unit vector from the *i*th grasshopper to the *j*th grasshopper and the social force $s(r)$ is defined by

3 $$s(r) = fe^{{ - \frac{r}{l}}} - e^{ - r} ,$$

where $f$ indicates the intensity of attraction and $l$ is the attractive length scale. In the Ref.^16^, $l = 1.5,f = 0.5.$

Let $d$ be the distance between two grasshoppers. $d=2.079$ is called the comfort zone or comfortable distance where there is neither attraction nor repulsion between two grasshoppers. When $d<2.079$, there is repulsion between two grasshoppers. When $d>2.079$, there is attraction between two grasshoppers. In particular, when $d$ changes from 2.079 to nearly 4, $s$ increases. When $d > 4$,$s$ decreases. When $d > 10$, $s$ trends to 0 and then $s$ has no action. Therefore, $d$ is mapped into the distance of grasshoppers in the interval of^1,4^. Thus the space between two grasshopper is divided into repulsion region, comfort zone, and attraction region.

$G_{i}$ in the Eq. (1) is defined by

4 $$G_{i} = - g\hat{e}_{i} ,$$

where $g$ is the gravitational constant and $\hat{e}_{i}$ is a unity vector towards the center of the earth.

$A_{i}$ in the Eq. (1) is defined by

5 $$A_{i} = u\hat{w}_{i} ,$$

where $u$ is a constant drift and $\hat{w}_{i}$ is a unity vector in the direction of the wind.

$S,G,A$ in the Eq. (1) are substituted by Eqs. (2)–(5) and the Eq. (1) becomes

6 $$X_{i} = \sum\limits_{j = 1,j \ne i}^{N} {s\left( {\left| {X_{j} - X_{i} } \right|} \right)} \frac{{X_{j} - X_{i} }}{{d_{ij} }} - g\hat{e}_{i} + u\hat{w}_{i} .$$

But the grasshoppers are as soon as located in the comfort zone and the swarm can not be converged into the appointed point. Therefore, Eq. (6) can not be used to solve the optimization model directly.

In order to solve the optimization model, the Eq. (6) is modified to be

7 $$X_{i}^{d} = c\sum\limits_{j = 1,j \ne i}^{N} {c\frac{{ub_{d} - lb_{d} }}{2}s\left( {\left| {X_{j}^{d} - X_{i}^{d} } \right|} \right)} \frac{{X_{j}^{d} - X_{i}^{d} }}{{d_{ij} }} + \hat{T}_{d} ,$$

where $ub_{d} ,lb_{d}$ are the upper bound and the lower bound of the *d*th component of the *i*th grasshopper, $\hat{T}_{d}$ is the $d$th component of the optimal grasshopper $\hat{T}$, the adaptive parameter $c$ is a decreasing coefficient to shrink the comfort zone, repulsion zone, and attraction zone. In Eq. (7), the gravity force is not considered, that is, there is no *G* component*.* And assume that the wind direction ($A$ component) is always towards a target $\hat{T}_{d}$.

In order to balance the exploration stage and the exploitation, the parameter $c$ is defined by

8 $$c = c_{\max } - t\frac{{c_{\max } - c_{\min } }}{T},$$

where $c_{\max } ,c_{\min }$ are the maximum and the minimum of the parameter $c$, respectively, $t$ denotes the current iteration and $T$ denotes the maximum iteration. In GOA, $c_{\max } = {1},c_{\min } = {0}{\text{.000001}}{.}$

### The improved GOA

Based on the influence of the gravity force not to be considered in the basic GOA, the right side of the Eq. (7) minus the sum of the product between the gravitational constant g and the unit vector from the *i*th grasshopper to the *j*th grasshopper, thus the new updated position of the grasshopper is obtained as follows

9 $$X_{i}^{d} = c\sum\limits_{j = 1,j \ne i}^{N} {c\frac{{ub_{d} - lb_{d} }}{2}s\left( {\left| {X_{j}^{d} - X_{i}^{d} } \right|} \right)} \frac{{X_{j}^{d} - X_{i}^{d} }}{{d_{ij} }} - \sum\limits_{j = 1,j \ne i}^{N} {g\frac{{X_{j}^{d} - X_{i}^{d} }}{{d_{ij} }}} + \hat{T}_{d} .$$

The velocity of the *i*th grasshopper causes its position updated during the hunting process as follows

10 $$v_{i}^{d} = cv_{i}^{d} + a \times rand \times (\hat{T}_{d} - X_{i}^{d} ),$$

11 $$X_{i}^{d} = X_{i}^{d} + v_{i}^{d} ,$$

where $a$ is the acceleration coefficient and $rand$ is the random number between 0 and 1.

Therefore, the *i*th grasshopper adopts two updated ways Eqs. (9) and (11) of the position. According to the selected probability $p$, the position of the *i*th grasshopper is updated as follows

12 $$X_{i}^{d} = \left\{ {\begin{array}{*{20}l} {c\sum\limits_{j = 1,j \ne i}^{N} {c\frac{{ub_{d} - lb_{d} }}{2}s\left( {\left| {X_{j}^{d} - X_{i}^{d} } \right|} \right)} \frac{{X_{j}^{d} - X_{i}^{d} }}{{d_{ij} }} - \sum\limits_{j = 1,j \ne i}^{N} {g\frac{{X_{j}^{d} - X_{i}^{d} }}{{d_{ij} }}} + \hat{T}_{d} ,} \hfill & {p < 0.5} \hfill \\ {X_{i}^{d} + cv_{i}^{d} + a \times rand \times (\hat{T}_{d} - X_{i}^{d} ),} \hfill & {p \ge 0.5} \hfill \\ \end{array} } \right.$$

where $c$ is the same as that in the basic GOA.

Based on the above, the GOA is improved, written as IGOA. The concrete steps of IGOA are as follows.

*Step 1*. Initialization. Initialize the grasshopper swarm $X_{i} (i = 1,2, \ldots ,N)$, $c_{\max } ,c_{\min }$, minimum and maximum of velocity, maximum number of iterations $T$.

*Step 2*. Calculate the fitness of every grasshopper and find the optimal grasshopper $\hat{T}$. Let $\text{t}=1.$

*Step 3*. Update the parameter $c$ by use of Eq. (8).

*Step 4*. For every grasshopper, the distance between the grasshoppers is firstly mapped into the interval^1,4^, and the selected probability $p$ is adopted. If $p < {0}{\text{.5}}$, the position of the grasshopper is updated by use of Eq. (12) or Eq. (9). Otherwise, the position of the grasshopper is updated by use of Eq. (12) or Eqs. (10) and (11). If the position of the grasshopper is out of the bound, then the position of the grasshopper is updated by use of the upper bound and the lower bound.

*Step 5*. Calculate the fitness value of every grasshopper. Update $\hat{T}$ if there is a better grasshopper. Let $t = t + 1$.

*Step 6*. Judge whether the terminal condition is satisfied. If YES, return the optimal grasshopper $\hat{T}$. Otherwise, turn Step 3.

## The function optimization

In this section, we adopt 23 benchmark functions to test the performance the proposed IGOA by compared with GOA, PSO, MFO, SCA, SSA, MVO and DA.

### 23 benchmark functions

**Figure 1:**
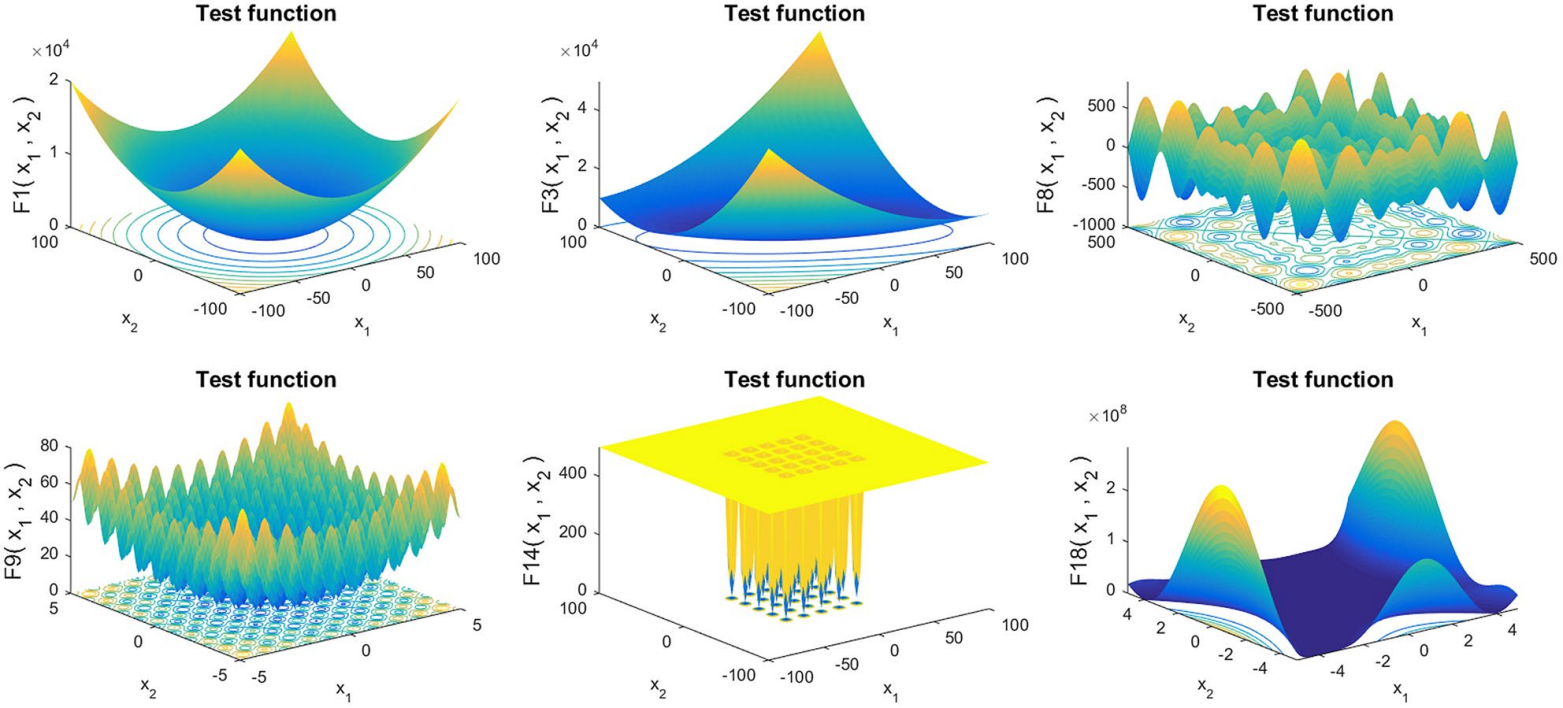
3D version of the benchmark functions $F_{{1}} (x),F_{3} (x),F_{8} (x),F_{9} (x),F_{14} (x),F_{{{18}}} (x)$ (using Matlab R2018a and www.mathworks.com).

In this subsection, 23 benchmark functions are derived from the Ref.^15^. Tables 1, 2 and 3 show the function expression, the dimension, the range and the minimum value of seven unimodal functions $F_{1} (x) - F_{7} (x)$ with $n$ dimension (Table 1), six multimodal functions $F_{{8}} (x) - F_{{{13}}} (x)$ with $n$ dimension (Table 2) and ten functions $F_{{{14}}} (x) - F_{{{23}}} (x)$ with the fixed dimension (Table 3), respectively. 3D version of some functions among these 23 benchmark functions are shown in Fig. 1.

**Table 1:** Unimodal functions $F_{1} (x) - F_{7} (x)$ with dimension $n$.

The function expression	Dim	Range	$f_{\min }$
$F_{1} (x) = \sum\limits_{i = 1}^{n} {x_{i}^{2} }$	30	[− 100, 100]	0
$F_{{2}} (x) = \sum\limits_{i = 1}^{n} {\left| {x_{i} } \right|} + \prod\limits_{i = 1}^{n} {\left| {x_{i} } \right|}$	30	[− 10, 10]	0
$F_{{3}} (x) = \sum\limits_{i = 1}^{n} {\left( {\sum\limits_{j = 1}^{i} {x_{j} } } \right)^{2} }$	30	[− 100, 100]	0
$F_{{4}} (x) = \mathop {{\text{max}}}\limits_{i} \left\{ {{\kern 1pt} \left| {x_{i} } \right|,1 \le i \le n} \right\}$	30	[− 100, 100]	0
$F_{5} (x) = \sum\limits_{i = 1}^{n - 1} {\left[ {100\left( {x_{i + 1} - x_{i}^{2} } \right)^{2} + \left( {x_{i} - 1} \right)^{2} } \right]}$	30	[− 30, 30]	0
$F_{6} (x) = \sum\limits_{i = 1}^{n} {\left( {\left\lfloor {x_{i} + 0.5} \right\rfloor } \right)^{2} }$	30	[− 100, 100]	0
$F_{7} (x) = \sum\limits_{i = 1}^{n} {ix_{i}^{4} } + random[0,1)$	30	[− 1.28, 1.28]	0

**Table 2:** Multimodal functions $F_{{8}} (x) - F_{{{13}}} (x)$ with dimension $n$.

The function expression	Dim	Range	$f_{\min }$
$F_{{8}} (x) = \sum\limits_{i = 1}^{n} {\left( { - x_{i} \sin \sqrt {\left| {x_{i} } \right|} } \right)}$	30	[− 500, 500]	$- 418.9829 \times {\text{Dim}}$
$F_{{9}} (x) = \sum\limits_{i = 1}^{n} {\left[ {x_{i}^{2} - 10\cos \left( {2\pi x_{i} } \right) + 10} \right]}$	30	[− 5.12, 15.12]	0
$$\begin{aligned} F_{{{10}}} (x) & = - 20\exp \left( { - 0.2\sqrt {\tfrac{1}{n}\sum\limits_{i = 1}^{n} {x_{i}^{2} } } } \right){\kern 1pt} - \exp \left( {\tfrac{1}{n}\sum\limits_{i = 1}^{n} {\cos (2\pi x_{i} )} } \right) \\ & \quad + 20 + \exp \\ \end{aligned}$$	30	[− 32, 32]	0
$F_{{{11}}} (x) = \tfrac{1}{4000}\sum\limits_{i = 1}^{n} {x_{i}^{2} } - \prod\limits_{i = 1}^{n} {\cos \tfrac{{x_{i} }}{\sqrt i }} + 1$	30	[− 600, 600]	0
$$\begin{aligned} & F_{{{12}}} (x) = \tfrac{\pi }{n}\left\{ {10\sin \left( {\pi y_{1} } \right) + \sum\limits_{i = 1}^{n - 1} {\left( {y_{i} - 1} \right)^{2} } [1 + 10\sin^{2} \left( {\pi y_{i + 1} } \right)]} \right. \\ & \quad \left. { + \left( {y_{n}^{2} - 1} \right)^{2} } \right\} + \sum\limits_{i = 1}^{n} {u(x_{i} ,10,100,4)} \\ & u(x_{i} ,a,k,m) = \left\{ {\begin{array}{*{20}l} {k(x_{i} - a)^{m} } \hfill & {x_{i} > a} \hfill \\ 0 \hfill & { - a < x_{i} < a} \hfill \\ {k( - x_{i} - a)^{m} } \hfill & {x < - a} \hfill \\ \end{array} } \right. \\ \end{aligned}$$	30	[− 50, 50]	0
$$\begin{aligned} F_{{{13}}} (x) & = {0}{\text{.1}}\left\{ {{\text{sin}}^{{2}} (3\pi x_{1} ) + \sum\limits_{i = 1}^{n} {(x_{i} - 1)^{2} [1 + \sin^{2} (3\pi x_{i} + 1)] + (x_{n} - 1)^{2} [1 + \sin^{2} (2\pi x_{n} )]} } \right\} \\ & \quad + \sum\limits_{i = 1}^{n} {u(x_{i} ,5,100,4)} \\ \end{aligned}$$	30	[− 50, 50]	0

**Table 3:** Benchmark functions $F_{{{14}}} (x) - F_{{{23}}} (x)$ with fixed dimension.

The function expression	Dim	Range	$f_{\min }$
$F_{{1{4}}} (x) = \left( {\frac{{1}}{{{500}}} + \sum\limits_{j = 1}^{25} {\frac{1}{{j + \sum\limits_{i = 1}^{2} {\left( {x_{i} - a_{ij} } \right)^{6} } }}} } \right)^{ - 1}$	2	[− 65.536, 65.536]	0.998
$F_{{{15}}} (x) = \sum\limits_{i = 1}^{{{11}}} {\left[ {a_{i} - \frac{{x_{1} (b_{i}^{2} + b_{i} x_{i} )}}{{b_{i}^{2} + b_{i} x_{3} + x_{4} }}} \right]^{2} }$	4	[− 5, 5]	0.0030
$F_{{{16}}} (x) = {4}x_{1}^{2} - 2.1x_{1}^{4} + \frac{1}{3}x_{1}^{6} + x_{1} x_{2} - 4x_{2}^{2} + 4x_{2}^{4}$	2	[− 5, 5]	− 1.0316
$F_{{{17}}} (x) = \left( {x_{2} - \frac{5.1}{{4\pi^{2} }}x_{1}^{2} + \frac{5}{\pi }x_{1} - 6} \right)^{2} + 10\left( {1 - \frac{1}{8\pi }} \right)\cos x_{1} + 10$	2	$[ - 5,10] \times [10,15]$	0.398
$$\begin{aligned} F_{{{18}}} (x) & = \left[ {{1} + \left( {x_{1} + x_{2} + 1} \right)^{2} \left( {19 - 14x_{1} + 3x_{1}^{2} - 14x_{2} + 6x_{1} x_{2} + 3x_{2}^{2} } \right)} \right] \\ & \quad \times \left[ {30 + \left( {2x_{1} - 3x_{2} } \right)^{2} \left( {18 - 32x_{1} + 12x_{1}^{2} + 48x_{2} - 36x_{1} x_{2} + 27x_{2}^{2} } \right)} \right] \\ \end{aligned}$$	2	[− 2, 2]	3
$F_{{{19}}} (x) = - \sum\limits_{i = 1}^{4} {c_{i} \exp \left( { - \sum\limits_{j = 1}^{3} {a_{ij} (x_{j} - p_{ij} )^{2} } } \right)}$	3	[0, 1]	− 3.86
$F_{{{20}}} (x) = - \sum\limits_{i = 1}^{4} {c_{i} \exp \left( { - \sum\limits_{j = 1}^{6} {a_{ij} (x_{j} - p_{ij} )^{2} } } \right)}$	6	[0, 1]	− 3.32
$F_{21} (x) = - \sum\limits_{i = 1}^{5} {\left[ {\left( {X - a_{i} } \right)\left( {X - a_{i} } \right)^{T} + c_{i} } \right]^{ - 1} }$	4	[0, 10]	− 10.1532
$F_{{2{2}}} (x) = - \sum\limits_{i = 1}^{{7}} {\left[ {\left( {X - a_{i} } \right)\left( {X - a_{i} } \right)^{T} + c_{i} } \right]^{ - 1} }$	4	[0, 10]	− 10.4028
$F_{{2{3}}} (x) = - \sum\limits_{i = 1}^{{{10}}} {\left[ {\left( {X - a_{i} } \right)\left( {X - a_{i} } \right)^{T} + c_{i} } \right]^{ - 1} }$	4	[0, 10]	− 10.5363

### The setup of the parameters

In order to verify the validation of the proposed IGOA in the paper, we choose GOA, PSO, MFO, SCA, SSA, MVO and DA to be the comparable algorithms. In the experiments, the parameters of these eight algorithms are set up as shown in Table 4.

**Table 4:** The setup parameters, where $t$ is the current iteration and $T$ is the maximal iteration.

Algorithm	Parameters	Value
IGOA	Adaptive parameter $c$	$c = c_{\max } - t\frac{{c_{\max } - c_{\min } }}{T}$
	Acceleration coefficient *a*	*a* = 2
	Gravitational constant $g$	$g = {0}{\text{.9}}$
GOA	Adaptive parameter $c$	$c = c_{\max } - t\frac{{c_{\max } - c_{\min } }}{T}$
PSO	Inertia weight $w$	$w = {1}$
	Acceleration coefficient $c_{{1}}$	$c_{{1}} = {2}$
	Acceleration coefficient $c_{{2}}$	$c_{{2}} = {2}$
MFO	Constant $b$ for defining the shape of the logarithmic spiral	$b = 1$
SCA	Random number $r_{1}$	$r_{1} = a - \frac{at}{T},a = 2$
SSA	Random number $c_{1}$	$c_{1} = 2e^{{ - \left( \frac{4t}{T} \right)^{2} }}$
MVO	Wormhole existence probability (WEP)	$WEP = \min + t \times \left( {\frac{\max - \min }{T}} \right),\min = 0.2,\max = 1$
	Travelling distance rate (TDR)	$TDR = 1 - \frac{{t^{{\tfrac{1}{p}}} }}{{T^{{\tfrac{1}{p}}} }},p = 6$
DA	Inertia weight $w$	$w = {\text{max}} - \frac{(\max - \min )t}{L},\min = 0.4,\max = 0.9$
	separation weight $s$	$s = {2} \times rand \times \left[ {0.1 - t\left( {\frac{0.1}{{L/2}}} \right)} \right]$
	alignment weight $a$	$a = {2} \times rand \times \left[ {0.1 - t\left( {\frac{0.1}{{L/2}}} \right)} \right]$
	the cohesion weight $c$	$c = {2} \times rand \times \left[ {0.1 - t\left( {\frac{0.1}{{L/2}}} \right)} \right]$
	food factor $f$	$s = {2} \times rand$
	enemy factor $e$	$s = 0.1 - t\left( {\frac{0.1}{{L/2}}} \right)$

### Experimental results

IGOA, GOA, PSO, MFO, SCA, SSA, MVO and DA all run 30 times independently. The average values and the standard deviations of the optimal function values of these 23 benchmark functions are obtained, shown in Table 5. In this section, we compare IGOA with GOA and PSO and then compare IGOA with MFO, SCA, SSA, MVO and DA.

**Table 5:** The average values and the standard deviations of the optimal function values of these 23 benchmark functions.

	IGOA		GOA		PSO		MFO		SCA		SSA		MVO		DA	
	Mean	Std	Mean	Std	Mean	Std	Mean	Std	Mean	Std	Mean	Std	Mean	Std	Mean	Std
${F}_{1}$*(x)*	4.9537E−03	6.2281E−03	3.1911E+01	2.0615E+01	4.9941E−01	1.4460E−01	2.6715E+03	5.2062E+03	1.1073E+01	1.1939E+01	**2.9954E−07**	6.0995E−07	1.3385E+00	4.1450E−01	9.9436E+00	1.8544E+01
${F}_{2}$*(x)*	2.9389E−01	1.8954E−01	1.6903E+01	2.1972E+01	4.8178E+00	1.5802E+00	3.5520E+01	1.8203E+01	**2.0518E−02**	2.9848E−02	2.0891E+00	1.5520E+00	1.3798E+01	3.1026E+01	1.3874E+00	9.3868E−01
${F}_{3}$*(x)*	**3.5429E−02**	3.0511E−02	3.2628E+03	1.6774E+03	1.2995E+03	1.9513E+03	1.8412E+04	1.1221E+04	9.0526E+03	6.9092E+03	1.3980E+03	7.8768E+02	2.1071E+02	9.9252E+01	2.4895E+02	3.4807E+02
${F}_{4}$*(x)*	**1.9125E−02**	1.3662E−02	1.3897E+01	3.7187E+00	2.5083E+00	1.2029E+00	7.0112E+01	7.3626E+00	3.7143E+01	1.1490E+01	1.1536E+01	4.7205E+00	2.1187E+00	8.0179E−01	2.5779E+00	1.5221E+00
${F}_{5}$*(x)*	**2.9610E**+**01**	8.3759E−01	5.0144E+03	7.5023E+03	2.0108E+02	1.6903E+02	2.6751E+06	1.4594E+07	1.3358E+05	3.8131E+05	3.6123E+02	6.0996E+02	5.6637E+02	8.1742E+02	5.3597E+03	1.3222E+04
${F}_{6}$*(x)*	2.0048E+00	5.6584E−01	5.0258E+01	3.9565E+01	7.0795E−01	2.2043E−01	2.3351E+03	5.0201E+03	1.4226E+01	2.3036E+01	**1.5109E−07**	2.0707E−07	1.1966E+00	4.2627E−01	9.7154E+00	1.5394E+01
${F}_{7}$*(x)*	**1.6040E−02**	1.2312E−02	4.8498E−02	1.9798E−02	2.6354E−01	1.1254E−01	4.9555E+00	6.8998E+00	8.6843E−02	7.5080E−02	1.7798E−01	8.9368E−02	3.5283E−02	1.4744E−02	3.2278E−02	2.1367E−02
${F}_{8}$*(x)*	− 7.1112E+03	6.9924E+02	− 7.3357E+03	7.0508E+02	− 2.9862E+03	3.5442E+02	− **8.5033E**+**03**	7.5759E+02	− 3.7147E+03	2.0745E+02	− 7.6155E+03	8.7803E+02	− 7.5698E+03	8.1819E+02	− 2.7765E+03	3.4388E+02
${F}_{9}$*(x)*	**4.2445E−01**	5.6806E−01	1.0498E+02	3.9353E+01	6.2949E+01	1.2413E+01	1.6249E+02	3.1801E+01	3.8650E+01	3.0163E+01	5.3927E+01	1.9322E+01	1.3209E+02	2.3029E+01	3.0467E+01	1.2955E+01
${F}_{10}$*(x)*	**5.2213E−02**	3.7227E−02	5.6079E+00	1.4100E+00	4.6567E+00	9.7259E−01	1.3410E+01	8.2129E+00	1.6576E+01	7.2415E+00	2.6304E+00	8.3827E−01	2.3565E+00	3.3404E+00	2.9176E+00	9.5108E−01
${F}_{11}$*(x)*	**2.9202E−04**	3.7925E−04	1.1091E+00	9.8949E−02	1.8703E+02	1.9145E+01	1.8909E+01	3.6619E+01	8.8654E−01	3.1146E−01	1.8252E−02	1.4655E−02	8.7141E−01	8.5692E−02	5.5753E−01	2.8875E−01
${F}_{12}$*(x)*	**2.2805E−01**	1.7421E−01	9.4889E+00	4.9347E+00	2.5243E+00	9.2169E−01	4.7287E+01	2.1718E+02	1.0826E+05	3.4685E+05	8.0007E+00	4.2546E+00	2.2880E+00	1.3491E+00	2.0946E+00	1.8474E+00
${F}_{13}$*(x)*	1.5727E+00	5.0315E−01	3.7212E+01	1.8566E+01	2.3815E+00	2.3544E+00	9.0138E+01	2.1255E+02	7.3125E+04	1.9072E+05	1.6785E+01	1.4144E+01	**1.7071E−01**	7.2391E−02	1.8396E+00	4.2620E+00
${F}_{14}$*(x)*	9.9816E−01	6.1346E−04	**9.9800E−01**	5.2481E−16	6.8263E+00	3.5355E+00	2.2837E+00	1.7232E+00	1.9204E+00	1.9052E+00	1.2626E+00	6.8541E−01	9.9800E−01	2.9891E−11	1.2295E+00	6.2117E−01
${F}_{15}$*(x)*	1.9673E−03	4.2459E−03	1.5969E−02	2.4649E−02	**6.3423E−04**	2.3951E−04	1.2850E−03	5.3964E−04	1.0897E−03	4.0441E−04	5.4929E−03	8.3801E−03	2.1203E−03	4.9657E−03	3.2414E−03	5.7635E−03
${F}_{16}$*(x)*	− 1.0313E+00	5.1361E−04	− 1.0316E+00	4.5530E−13	− 1.0316E+00	6.5216E−05	− **1.0316E+00**	0.0000E+00	− 1.0316E+00	3.3087E−05	− 1.0316E+00	2.5447E−14	− 1.0316E+00	3.1609E−07	− 1.0316E+00	5.0989E−06
${F}_{17}$*(x)*	3.9851E−01	9.9557E−04	3.9789E−01	2.1858E−12	3.9814E−01	4.8925E−04	**3.9789E−01**	0.0000E+00	4.0187E−01	9.1717E−03	3.9789E−01	7.7808E−14	3.9789E−01	1.0016E−06	3.9789E−01	2.2095E−07
${F}_{18}$*(x)*	3.0152E+00	1.2467E−02	5.7000E+00	1.4789E+01	3.0075E+00	7.3803E−03	**3.0000E+00**	2.9995E−15	3.0001E+00	7.5162E−05	3.0000E+00	3.0698E−13	3.0000E+00	3.0522E−06	3.0000E+00	6.3603E−05
${F}_{19}$*(x)*	− 3.8583E+00	4.3561E−03	− 3.7901E+00	1.6365E−01	− 3.8614E+00	9.7738E−04	− **3.8628E+00**	2.7101E−15	− 3.8533E+00	3.0233E−03	− 3.8628E+00	4.2796E−10	− 3.8628E+00	1.6161E−06	− 3.8627E+00	1.0680E−04
${F}_{20}$*(x)*	− 3.2475E+00	6.9085E−02	− 3.2759E+00	6.1787E−02	− 3.0970E+00	3.1959E−01	− 3.2234E+00	5.8608E−02	− 2.9672E+00	2.6799E−01	− 3.2126E+00	5.8889E−02	− **3.2858E+00**	5.6233E−02	− 3.2504E+00	9.3598E−02
${F}_{21}$*(x)*	− **8.9507E+00**	1.8094E+00	− 4.9683E+00	3.0739E+00	− 5.1417E+00	2.9174E+00	− 6.2239E+00	3.3915E+00	− 2.5289E+00	1.7852E+00	− 7.8105E+00	3.2181E+00	− 7.2965E+00	3.2182E+00	− 7.1090E+00	2.7674E+00
${F}_{22}$*(x)*	− **9.4709E+00**	1.4775E+00	− 5.3995E+00	3.4978E+00	− 6.0982E+00	3.5458E+00	− 7.5677E+00	3.5774E+00	− 3.0696E+00	1.7428E+00	− 8.1241E+00	3.3366E+00	− 8.3030E+00	2.8607E+00	− 6.7733E+00	2.9161E+00
${F}_{23}$*(x)*	− **9.3486E+00**	1.9572E+00	− 6.2373E+00	3.8686E+00	− 6.1745E+00	3.7598E+00	− 7.9274E+00	3.5389E+00	− 3.7453E+00	1.5528E+00	− 8.1935E+00	3.4369E+00	− 8.7642E+00	3.0618E+00	− 7.6592E+00	3.4776E+00

The bold indicates the optimal value for each benchmark function.

#### IGOA vs. (GOA, PSO)

For the unimodal functions $F_{1} (x) - F_{{5}} (x),F_{7} (x)$, the average values of the optimal values obtained by IGAO are 4.9537E−03, 2.9389E−01, 3.5429E−02, 1.9125E−02, 2.9610E+01 and 1.6040E−02, respectively, which are all less than those obtained by GOA and PSO. But for the unimodal function $F_{{6}} (x)$, the average value of the optimal value obtained by IGAO is 2.0048E+00, which is less than that obtained by GOA and is more than that obtained by PSO.

For the multimodal functions $F_{{9}} (x) - F_{{{13}}} (x)$, the average values of the optimal values obtained by IGAO are 4.2445E−01, 5.2213E−02, 2.9202E−04, 2.2805E−01 and 1.5727E+00, respectively, which are all less than those obtained by GOA and PSO. But for the multimodal function $F_{{8}} (x)$, the average value of the optimal value obtained by IGAO is − 7.1112E+03, which is more than that obtained by GOA and is less than that obtained by PSO.

For the functions $F_{{{14}}} (x),F_{20} (x)$, the average values of the optimal values obtained by IGAO are 9.9816E−01 and − 3.2475E+00, respectively, whose degree closest to the optimal values 9.9800E−01 and − 3.32 is less than those obtained by GOA and is more than those obtained by PSO. For the functions $F_{{{16}}} (x) - F_{17} (x)$, the average values of the optimal values obtained by IGAO are − 1.0313E+00 and 3.9851E−01, whose degrees closest to the optimal values − 1.0316E+00 and 0.3978 are all less than GOA and PSO. For the functions $F_{{{18}}} (x) - F_{19} (x)$, the average values of the optimal values obtained by IGAO are 3.0152E+00 and − 3.8583E+00, whose degree closest to the optimal values 3 and − 3.86 is less than that obtained by PSO and is more than that obtained by GOA. For the functions $F_{15} (x),F_{{{21}}} (x) - F_{{{23}}} (x)$, the average values of the optimal values obtained by IGAO are 1.9673E−03, − 8.9507E+00, − 9.4709E+00 and − 9.3486E+00, whose degrees closest to the optimal values 0.0030, − 10.1532, − 10.4028 and − 10.5363 are more than those obtained by GOA and PSO.

**Figure 2:**
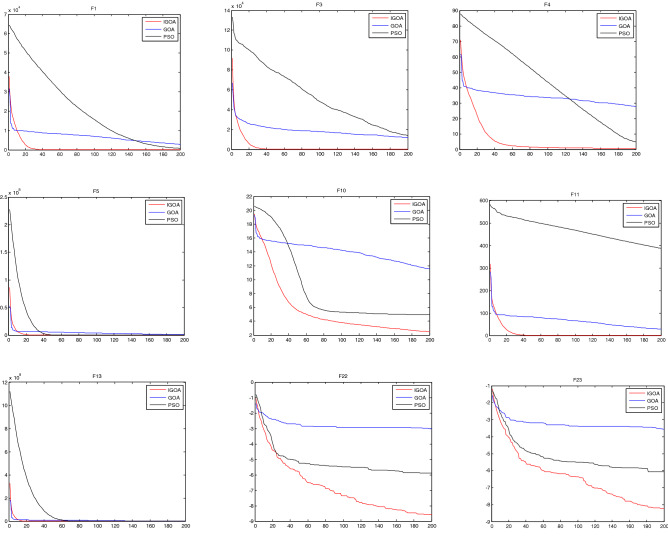
The convergence curves of the functions $F_{{1}} (x),F_{3} (x) - F_{5} (x),F_{10} (x) - F_{11} (x),F_{13} (x),F_{22} (x) - F_{23} (x).$

Figure 2 shows the convergence curves of IGOA, GOA and PSO on the first 200 iterations on benchmark functions $F_{{1}} (x),F_{3} (x) - F_{5} (x),F_{10} (x) - F_{11} (x),F_{13} (x),F_{22} (x) - F_{23} (x).$ It is observed from Fig. 2 that the convergence speed of IGOA is faster than those of GOA and PSO. Table 5 and Fig. 2 show that IGOA outperforms GOA and PSO.

#### IGOA vs. (MFO, SCA, SSA, MVO, DA)

For the unimodal functions $F_{1} (x),F_{6} (x)$, the average values of the optimal values obtained by IGAO are 4.9537E−03 and 2.0048E+00, which are less than those obtained by MFO, SCA, MVO and DA and are more than those obtained by SSA. For the unimodal function $F_{2} (x)$, the average value of the optimal value obtained by IGAO is 2.9389E−01, which is less than that obtained by MFO, SSA, MVO and DA and is more than that obtained by SCA. For the unimodal functions $F_{3} (x) - F_{{5}} (x),F_{7} (x)$, the average values of the optimal values obtained by IGAO are 3.5429E−02, 1.9125E−02, 2.9610E+01 and 1.6040E−02, less than those obtained by MFO, SCA, SSA, MVO and DA.

For the multimodal functions $F_{{9}} (x) - F_{{{13}}} (x)$, the average values of the optimal values obtained by IGAO are 4.2445E−01, 5.2213E−02, 2.9202E−04, 2.2805E−01 and 1.5727E+00, which are all less than those obtained by MFO, SCA, SSA, MVO and DA. For the multimodal function $F_{{8}} (x)$, the average value of the optimal value obtained by IGAO is − 7.1112E+03, which is more than that obtained by SCA, SSA, MVO and DA and is less than that obtained by MFO.

For the function $F_{{{14}}} (x)$, the average value of the optimal value obtained by IGAO is 9.9816E−01, whose degree closest to the optimal value 9.9800E−01 is less than that obtained by MVO and is more than that obtained by MFO, SCA, SSA and DA. For the function $F_{{{15}}} (x)$, the average value of the optimal value obtained by IGAO is 1.9673E−03, whose degree closest to the optimal value 0.0030 is less than that obtained by MVO and DA and is more than that obtained by MFO, SCA and SSA. For the function $F_{{{16}}} (x)$, the average value of the optimal value obtained by IGAO is − 1.0313E+00, whose degree closest to the optimal value − 1.0316E+00 is less than that obtained by MFO, SCA, SSA, MVO and DA. For the function $F_{{{17}}} (x)$, the average value of the optimal value obtained by IGAO is 3.9851E−01, whose degree closest to the optimal value 0.398 is less than that obtained by MFO, SSA, MVO and DA and is more than that obtained by SCA. For the function $F_{{{18}}} (x)$, the average value of the optimal value obtained by IGAO is 3.0152E+00, whose degree closest to the optimal value 3 is less than that obtained by MFO, SCA, SSA, MVO and DA. For the function $F_{{{20}}} (x)$, the average value of the optimal value obtained by IGAO is − 3.2475E+00, whose degree closest to the optimal value − 3.32 is less than that obtained by MVO and is more than that obtained by MFO, SCA, SSA and DA. For the functions $F_{19} (x),F_{{{21}}} (x) - F_{{{23}}} (x)$,the average values of the optimal values obtained by IGAO are − 3.8583E+00, − 8.9507E+00, − 9.4709E+00 and − 9.3486E+00, which is the closer to the optimal values − 3.86, − 10.1532, − 10.4028 and − 10.5363 than those obtained by MFO, SCA, SSA, MVO and DA.

Therefore, it is observed from Table 5 that IGOA outperforms MFO, SCA, SSA, MVO and DA. By the comparison in the “IGOA vs. (GOA, PSO)” and “IGOA vs. (MFO, SCA, SSA, MVO, DA)”, IGOA can be used to perform the function optimization and is superior to GOA, PSO, MFO, SCA, SSA, MVO and DA, which is utilized to verify no free lunch theorem.

## Applications

In this section, the proposed IGOA is used to optimize the weights and the bases of the BP neural network, and then predicted model IGOA-BPNN is obtained. Finally, the IGOA-BPNN is used to perform the prediction of the closing prices of the Shanghai Stock Exchange Index and the air quality index (AQI) of Taiyuan, Shanxi Province.

### Predicted model

IGOA proposed in this paper is used to optimize the connected weights and the bases of BP neural network, and the predicted model IGOA-BPNN is established. The concrete steps of IGOA-BPNN are as follows:

*Step 1*: Input data and divide these data into the training set and the testing data. Normalize the training set and the testing set.

*Step 2*: Initialize the size of the grasshopper swarm, $c_{\max } ,c_{\min }$, minimum and maximum of velocity, the maximum number of iterations of IGOA, and the number of the nodes in the hidden layer of BP neural network and the maximum number of iterations of BP neural network. Choose Mean Square Error (MSE)

13 $$MSE = \frac{1}{Q}\sum\limits_{s = 1}^{Q} {\left( {y_{s} - t_{s} } \right)^{2} }$$

to be the fitness function of IGOA, where $y_{s} ,t_{s}$ are the predicted value and the target value of the $s$th sample. Let $t = 1$.

*Step 3*: Initialize the grasshopper swarm.

*Step 4*: Map every grasshopper into the connected weights between the input layer and the hidden layer, the bases in the hidden layer, the connected weights between the hidden layer and the output layer, the bases in the output layer of BP neural network. Then train BP neural network and obtain the predicted values of the training set. According to predicted values and the target values, calculate the fitness value of this grasshopper. Find out the optimal grasshopper.

*Step 5*: Update the grasshopper swarm by use of IGOA. Then let $t = t + 1$.

*Step 6*: If the termination conditions are satisfied, then turn Step 6, otherwise turn Step 3.

*Step 7*: Output the optimal grasshopper. Then map the optimal grasshopper into the connected weights between the input layer and the hidden layer, the bases in the hidden layer, the connected weights between the hidden layer and the output layer, the bases in the output layer of BP neural network. Then train BP neural network and obtain the predicted values of the training set. Thus obtain the trained BP neural network. Finally input the testing set into the trained BP neural network and obtain the predicted values of the testing set.

**Figure 3:**
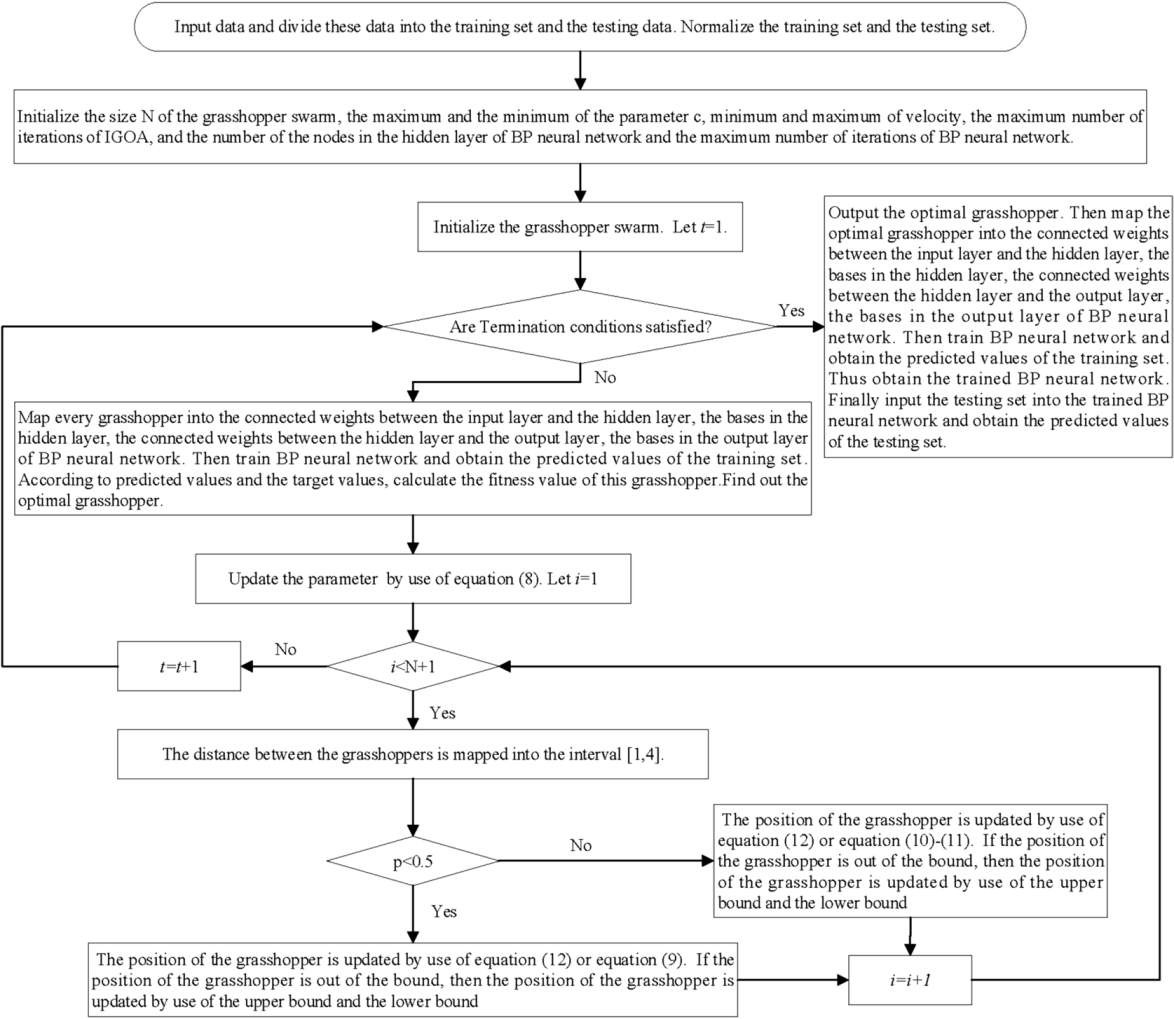
The flowchart of IGOA-BPNN.

The flowchart of IGOA-BPNN is shown in Fig. 3.

In this section, MSE, Mean Absolute Error (MAE)

14 $$MAE = \frac{1}{Q}\sum\limits_{i = 1}^{Q} {\left| {y_{s} - t_{s} } \right|} ,$$

Root Mean Square Error (RMSE)

15 $$RMSE = \frac{1}{Q}\sqrt {\sum\limits_{i = 1}^{Q} {\left( {y_{s} - t_{s} } \right)^{2} } } ,$$

Mean Absolute Percentage Error (MAPE)

16 $$MAPE = \frac{1}{Q}\sum\limits_{i = 1}^{Q} {\frac{{\left| {y_{s} - t_{s} } \right|}}{{\left| {t_{s} } \right|}}} \times 100\% ,$$

are taken to the evaluation criteria of model, where $y_{s} ,t_{s}$ are the predicted value and the target value of the $s$th sample.

### Prediction of Shanghai Stock Index

#### Data source

**Figure 4:**
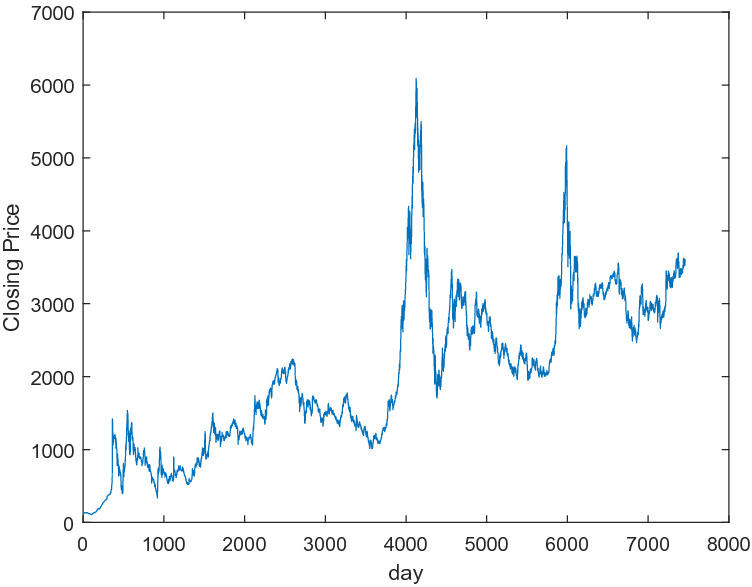
The trends of the 7459 days’ closing prices of Shanghai Stock Index.

In this subsection, Shanghai Stock 000001 from December 19, 1990 to June 28, 2021 loaded from the website http://quotes.money.163.com/trade/lsjysj_zhishu_000001.html contains 7459 days’ data. Figure 4 shows that the trend of the 7459 days’ closing prices. The features of every data sample consist of the closing price, the highest price, the lowest price, the opening price, the previous closing, the rise and fall amount, the rise and fall range, the trading volume and the transaction amount. In this paper, we choose the closing price, the highest price, the lowest price, the opening price, the previous closing, the trading volume and the transaction amount to be the features of samples and use the features of the current day to predict the closing price of the next day. The 7383 data samples from December 19, 1990 to March 5, 2021 are taken to the training set and the remaining 75 data samples from March 6, 2021 to June 27, 2021 are taken to the testing set.

#### Experimental results

In this subsection, we use the predicted model IGOA-BPNN to predict the closing price of Shanghai Stock Index 000001. In order to verify the performance of the predicted model IGOA-BPNN, GOA, PSO, MFO, SCA, SSA, MVO and DA are employed to be combined with BP neural network (BPNN) to establish the comparable predicted models GOA-BPNN, PSO-BPNN, MFO-BPNN, SCA-BPNN, SSA-BPNN, MVO-BPNN and DA-BPNN, respectively. Meantime, BPNN is also employed to be the comparable predicted model.

Among the BPNN and the BPNN part of these comparable predicted models IGOA-BPNN, GOA-BPNN, PSO-BPNN, MFO-BPNN, SCA-BPNN, SSA-BPNN, MVO-BPNN and DA-BPNN, the maximum number of iterations is set up to be 5000 and the momentum factor is 0.95. IGOA-BPNN, GOA-BPNN, PSO-BPNN, MFO-BPNN, SCA-BPNN, SSA-BPNN, MVO-BPNN, DA-BPNN and BPNN are run independently 30 times, respectively, and then MSE, MAE, RMSE, MAPE of the average predicted values of these 75 testing samples are obtained, shown in Table 6.

**Table 6:** The predicted errors of the testing samples on Shanghai Stock Index.

	IGOA-BPNN	GOA-BPNN	PSO-BPNN	MFO-BPNN	SCA-BPNN	SSA-BPNN	MVO-BPNN	DA-BPNN	BPNN
*MSE*	**828.95**	875.57	858.88	854.16	850.04	850.04	880.46	895.94	1255.85
*MAE*	**21.70**	22.56	21.93	22.39	23.35	22.29	22.47	22.75	28.54
RMSE	**3.32**	3.42	3.38	3.37	3.53	3.37	3.43	3.46	4.09
*MAPE(%)*	**0.62**	0.65	0.63	0.64	0.67	0.64	0.65	0.66	0.82

The bold indicates the minimum error.

**Figure 5:**
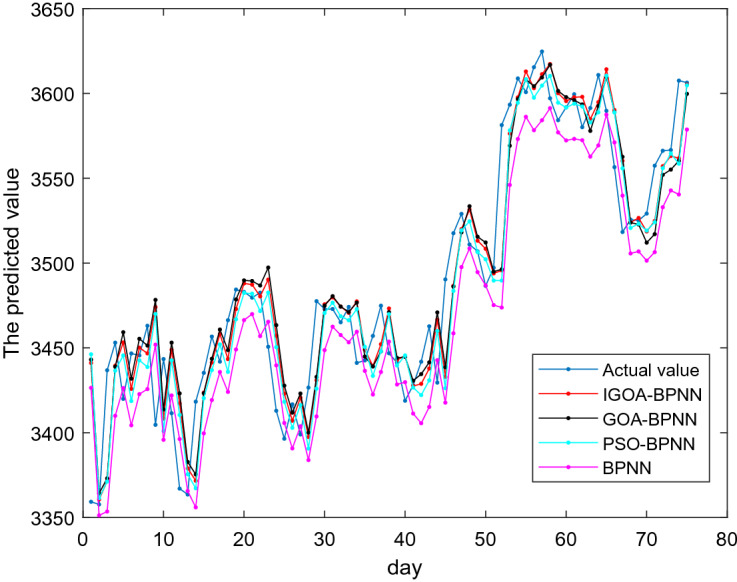
Comparison among the actual values and the predicted values of four models.

Figure 5 shows the comparison among the actual values and the predicted values of IGOA-BPNN, GOA-BPNN, PSO-BPNN and BPNN of these 75 testing samples.

From Table 6, the predicted errors *MSE* = 828.95, *MAE* = 21.70, *RMSE* = 3.32, *MAPE* = 0.62% obtained from predicted model IGOA-BPNN are less than those obtained from the predicted models GOA-BPNN, PSO-BPNN, MFO-BPNN, SCA-BPNN, SSA-BPNN, MVO-BPNN, DA-BPNN and BPNN, which shows that the proposed IGOA in this paper is more suitable for optimizing the parameters of BP neural network for predicting Shanghai Stock Index 000001. It is also observed from Table 6 that the predicted performance of the BP neural network optimized by swarm intelligence algorithms outperforms the pure BP neural network.

### Prediction of air quality index in Taiyuan, Shanxi

#### Data source

**Figure 6:**
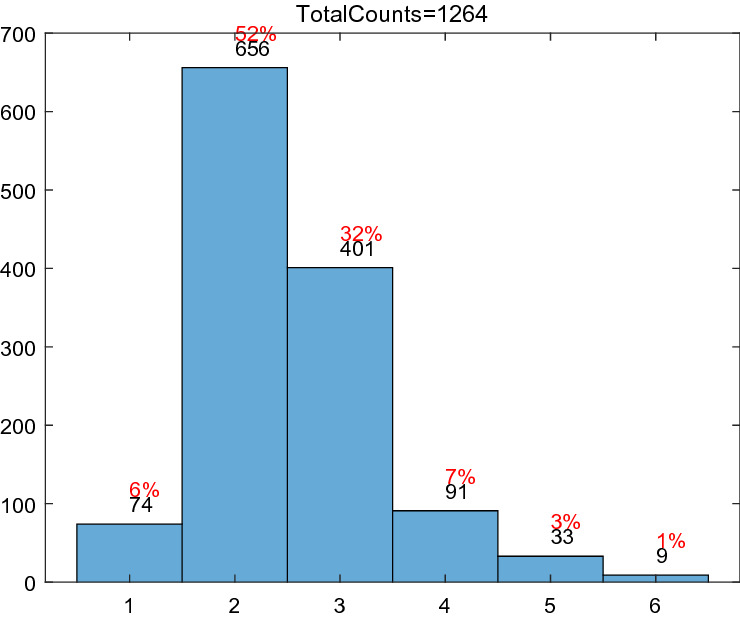
The number of days and proportion distribution of six grades.

**Figure 7:**
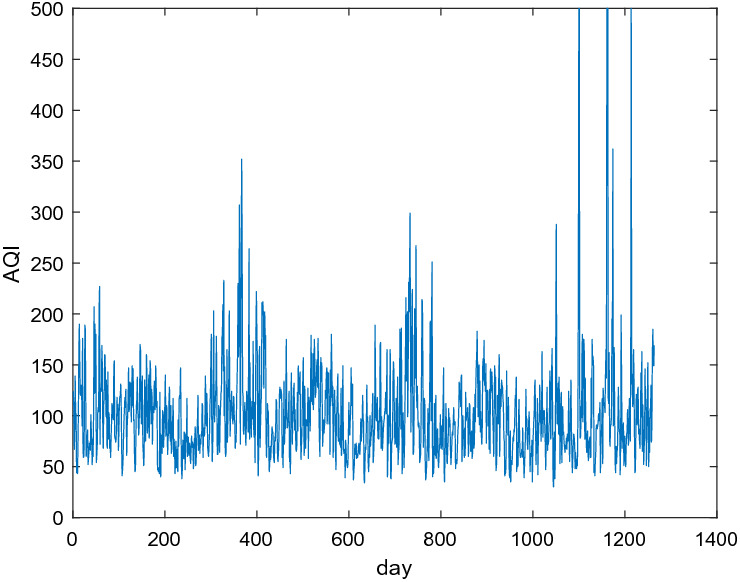
Trends of AQI in these 1264 days.

The 1273 day’s data from January 1, 2018 to June 26, 2021 in this subsection is derived from the website https://www.aqistudy.cn/historydata/monthdata.php?city=%e5%a4%aa%e5%8e%9f. The features of every data consist of the current air quality index (AQI), quality grade, PM2.5, PM10, SO_2_, CO, NO_2_, O_3_. The relation between AQI and quality grade is shown in Table 7. The remaining 1264 data samples are kept by deleting the missing data. Figure 6 shows the number of days and proportion distribution of excellent, good, light pollution, moderate pollution, heavy pollution and serious pollution in these 1264 days. Figure 7 shows the trends of AQI in these 1264 days.

**Table 7:** The relation of AQI and quality grade.

Range of AQI	0–50	51–100	101–150	151–200	201–300	> 300
Quality grade	Excellent	Good	Light pollution	Moderate pollution	Severe pollution	Serious pollution

In this subsection, we choose the current AQI, PM2.5, PM10, SO_2_, CO, NO_2_ and O_3_ to predict AQI of the next day and select the 1241 data samples from January 1, 2018 to June 3, 2021 to be training set and the 22 data samples from June 4, 2021 to June 25,2021 to be the testing set.

#### Experimental results

**Figure 8:**
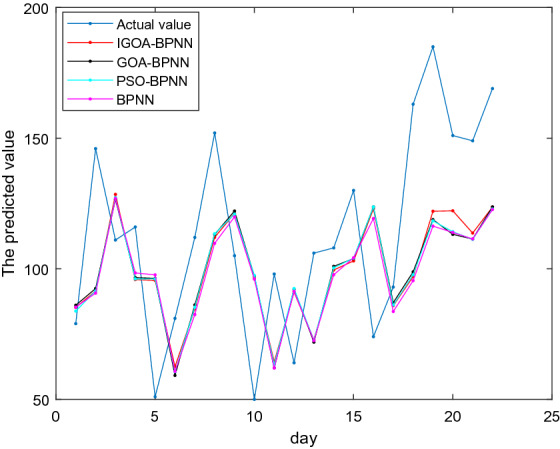
Comparison among the actual values and the predicted values of four models.

The proposed IGOA-BPNN in this subsection is utilized to predict AQI in Taiyuan, Shanxi. Similar to “Experimental results”, among the BPNN and the BPNN part of these comparable predicted models IGOA-BPNN, GOA-BPNN, PSO-BPNN, MFO-BPNN, SCA-BPNN, SSA-BPNN, MVO-BPNN and DA-BPNN, the maximum number of iterations is set up to be 5000 and the momentum factor is 0.95. IGOA-BPNN, GOA-BPNN, PSO-BPNN, MFO-BPNN, SCA-BPNN, SSA-BPNN, MVO-BPNN, DA-BPNN and BPNN are run independently 10 times, respectively, and then MSE, MAE, RMSE, MAPE of the average predicted values of these 22 testing samples are obtained, shown in Table 8. Figure 8 shows the comparison among the actual values and the predicted values of IGOA-BPNN, GOA-BPNN, PSO-BPNN and BPNN of these 22 testing samples.

**Table 8:** Prediction errors of AQI test samples in Taiyuan, Shanxi.

	IGOA-BPNN	GOA-BPNN	PSO-BPNN	MFO-BPNN	SCA-BPNN	SSA-BPNN	MVO-BPNN	DA-BPNN	BPNN
*MSE*	**1340.43**	1392.94	1399.39	1376.57	1352.42	1406.43	1451.88	1373.48	1433.37
*MAE*	**32.51**	33.17	33.13	32.91	32.75	33.14	33.84	32.81	33.61
RMSE	**7.81**	7.96	7.98	7.91	7.84	8.00	8.12	7.90	8.07
*MAPE(%)*	**31.91**	32.58	32.46	32.10	32.14	32.44	32.94	32.17	32.71

The bold indicates the minimum error.

From Table 8, the predicted errors *MSE* = 134.43, *MAE* = 32.51, *RMSE* = 7.81, *MAPE* = 31.91% obtained from predicted model IGOA-BPNN are less than those obtained from the predicted models GOA-BPNN, PSO-BPNN, MFO-BPNN, SCA-BPNN, SSA-BPNN, MVO-BPNN, DA-BPNN and BPNN, which shows that the proposed IGOA in this paper is more suitable for optimizing the parameters of BP neural network for predicting AQI in Taiyuan, Shanxi. It is also observed from Table 8 that the predicted performance of the BP neural network optimized by swarm intelligence algorithms outperforms the pure BP neural network.

## Conclusions and discussion

The updated methods of velocity and position of PSO are introduced into GOA and thus improved GOA is obtained, written as IGOA. Then 23 benchmark functions are used to verify the effectiveness of IGOA, and the experimental results show that IGAO is superior to MFO, SCA, SSA, MVO and DA. Finally IGOA is utilized to optimize the connection weight and bases of BP neural network, and the prediction model IGOA-BPNN is established. IGOA-BPNN is applied to the prediction of Shanghai stock index and AQI of Taiyuan, Shanxi. The results show that IGOA-BPNN is better than GOA-BPNN, PSO-BPNN, MFO-BPNN, SCA-BPNN, SSA-BPNN, MVO-BPNN, DA-BPNN and BPNN.

However, the size of the initial population of IGOA, the parameters of BP neural network, and other machine learning methods may lead to different experimental results. Therefore, it is necessary to study the combination of swarm intelligence algorithm and different machine learning to establish prediction models to solve practical problems.
